# Mining Online Social Network Data for Biomedical Research: A Comparison of Clinicians’ and Patients’ Perceptions About Amyotrophic Lateral Sclerosis Treatments

**DOI:** 10.2196/jmir.2127

**Published:** 2012-06-21

**Authors:** Carlos Nakamura, Mark Bromberg, Shivani Bhargava, Paul Wicks, Qing Zeng-Treitler

**Affiliations:** ^1^Department of Biomedical InformaticsUniversity of UtahSalt Lake City, UTUnited States; ^2^Department of NeurologyUniversity of UtahSalt Lake City, UTUnited States; ^3^PatientsLikeMeCambridge, MAUnited States

**Keywords:** Amyotrophic lateral sclerosis, ALS, Lou Gehrig’s disease, treatment response, treatment efficacy, online social network, online patient community

## Abstract

**Background:**

While only one drug is known to slow the progress of amyotrophic lateral sclerosis (ALS), numerous drugs can be used to treat its symptoms. However, very few randomized controlled trials have assessed the efficacy, safety, and side effects of these drugs. Due to this lack of randomized controlled trials, consensus among clinicians on how to treat the wide range of ALS symptoms and the efficacy of these treatments is low. Given the lack of clinical trials data, the wide range of reported symptoms, and the low consensus among clinicians on how to treat those symptoms, data on the prevalence and efficacy of treatments from a patient’s perspective could help advance the understanding of the symptomatic treatment of ALS.

**Objective:**

To compare clinicians’ and patients’ perspectives on the symptomatic treatment of ALS by comparing data from a traditional survey study of clinicians with data from a patient social network.

**Methods:**

We used a survey of clinicians’ perceptions by Forshew and Bromberg as our primary data source and adjusted the data from PatientsLikeMe to allow for comparisons. We first extracted the 14 symptoms and associated top four treatments listed by Forshew and Bromberg. We then searched the PatientsLikeMe database for the same symptom–treatment pairs. The PatientsLikeMe data are structured and thus no preprocessing of the data was required.

**Results:**

After we eliminated pairs with a small sample, 15 symptom–treatment pairs remained. All treatments identified as useful were prescription drugs. We found similarities and discrepancies between clinicians’ and patients’ perceptions of treatment prevalence and efficacy. In 7 of the 15 pairs, the differences between the two groups were above 10%. In 3 pairs the differences were above 20%. Lorazepam to treat anxiety and quinine to treat muscle cramps were among the symptom–treatment pairs with high concordance between clinicians’ and patients’ perceptions. Conversely, amitriptyline to treat labile emotional effect and oxybutynin to treat urinary urgency displayed low agreement between clinicians and patients.

**Conclusions:**

Assessing and comparing the efficacy of the symptomatic treatment of a complex and rare disease such as ALS is not easy and needs to take both clinicians’ and patients’ perspectives into consideration. Drawing a reliable profile of treatment efficacy requires taking into consideration many interacting aspects (eg, disease stage and severity of symptoms) that were not covered in the present study. Nevertheless, pilot studies such as this one can pave the way for more robust studies by helping researchers anticipate and compensate for limitations in their data sources and study design.

## Introduction

Clinical trials, surveys, and medical records are conventional sources of data for medical research. Some researchers have begun to explore alternative data sources to better understand the patient’s perspective [1]. An example of such alternative data sources is online social networks such as PatientsLikeMe [2]. In recent years, online social network sites with a health focus have attracted large numbers of users and have begun accumulating large quantities of detailed clinical information. Information gathered by these networks is primarily intended for patients to share with each other. However, such information has also begun to attract the attention of medical researchers for a variety of uses including clinical trial recruitment [3], development of patient-reported outcomes [4,5], and perhaps even novel ways of evaluating treatment efficacy [6-8].

Because social networks are a relatively new phenomenon, their strengths and limitations as a data source for medical research have not been carefully investigated. On the one hand, the large amount of patient data on social networks could bring a great contribution to studies on important issues such as treatment response and compliance. On the other hand, we need to understand whether and how these data provide us with additional information in comparison with traditional data sources, as well as the validity of that information.

In this study, we proposed to compare clinicians’ and patients’ perspectives on the symptomatic treatment of amyotrophic lateral sclerosis (ALS) by comparing data from a traditional survey study of clinicians with data from a patient social network. Forshew and Bromberg extracted data on clinicians’ perspectives from a national survey among ALS clinical centers [9]. Data on patients’ perspectives were extracted from the online social network PatientsLikeMe.

ALS, also known as Lou Gehrig’s disease, is a rare but fatal neurodegenerative disease that causes muscle weakness. Most patients die of respiratory failure within 3 to 5 years of the disease onset [10].

While only one drug is known to slow the progress of ALS (riluzole), numerous drugs can be used to treat its diverse array of symptoms. However, very few randomized controlled trials have assessed the efficacy, safety, and side effects of potential treatments to modulate these symptoms. Consensus among clinicians on how to treat the wide range of ALS symptoms and the efficacy of these treatments have historically been low [9]. The American Academy of Neurology, in developing their recent guidelines on the management of ALS [11], found only limited evidence to support treatment choice for symptom management. Evidence was most robust for treatment of excessive saliva (level B evidence for botox, level C evidence for irradiation of the salivary gland) and pseudobulbar affect (level B evidence for the efficacy of dextromethorphan and quinidine), but wholly lacking for treatment of fatigue (level C evidence for withdrawing riluzole), cramps (level U, no appropriate studies), spasticity (level U), depression (level U), and anxiety (level U). Given (1) the lack of evidence to support decisions (2), the wide range of reported symptoms, and (3) the low consensus among clinicians on how to treat those symptoms, data on the prevalence and efficacy of treatments from a patient’s perspective could help advance the understanding of the symptomatic treatment of ALS.

### Background

One approach to assessing the efficacy of symptomatic drugs when randomized controlled trials are lacking is through a survey instrument fielded to clinicians, patients, or caregivers. In 2003, Forshew and Bromberg [9] reported the results of a national survey sent out to 61 ALS clinics in North America, to which 39 clinics replied. Clinicians were asked to name the three most frequent interventions they would use for each symptom and their respective rankings for their perception of efficacy in relieving the symptom (ranging from 1, rarely effective, to 5, nearly complete relief). The 14 symptoms included in the survey were selected based on a literature search. The article reported the four top interventions for each of the predetermined symptoms.

Forshew and Bromberg mentioned in their article that a mirror study giving the patients’ perspective on the same topic was underway, though the results of that study have not been published yet. In the absence of such a study, it is still possible to contrast clinicians’ and patients’ perspectives by looking into rapidly increasing and publicly available databases of online communities of patients. PatientsLikeMe is an online community that allows members to track their progress with clinical scales, share information, and learn more about their condition. It has established data-sharing partnerships with doctors, pharmaceutical and medical device companies, research organizations, and nonprofits. Starting with an ALS patient community, PatientsLikeMe now hosts communities for more than 100,000 patients with any medical condition. Currently, the US ALS user base represents about 5% of the US ALS population and is an almost perfect match in terms of demographics, but with a bias toward long-term survivors. By definition, a bias toward patients with computer and Web access is indeed also present.

Each community member is asked to track 11 to 12 primary symptoms of his or her condition. The primary symptom list was generated for the ALS community with input from health care practitioners and the literature. Members can also report, in free-text form, any additional symptoms they are experiencing. The result is a semistructured list, which patients can use as an aid for future symptom reporting. It also permits comparison with symptoms reported by other patients in the system. Additional symptoms entered by a member lead to a prompt (“did you mean...”) to attempt to merge their description with existing symptom entries; if they wish to add a new symptom term, this is reviewed by a nurse curator.

Comparing data from very different sources is challenging, as the many adjustments that datasets must go through may induce further biases. However, in the absence of robust evidence from randomized controlled trials (which also have their own limitations and weaknesses), the data from surveys and online communities of patients become a viable resource in the search for a better understanding of clinicians’ and patients’ perceptions.

## Methods

### Treatment Prevalence

We used the Forshew and Bromberg study as our primary data source and adjusted the data from PatientsLikeMe to allow for comparisons. We first extracted the 14 symptoms and associated top four treatments listed by Forshew and Bromberg. We then searched the PatientsLikeMe database for the same symptom–treatment pairs. Members of PatientsLikeMe also mentioned 13 of the 14 symptoms listed by Forshew and Bromberg. The only nonoverlapping symptom was thick phlegm, which had no corresponding item. The PatientsLikeMe data are structured and thus no preprocessing of the data was required.

### Treatment Efficacy

In this study we proposed to compare clinicians’ and patients’ perceptions of the efficacy of different treatments to alleviate ALS symptoms. There is one important distinction between the perceptions of these two groups. Patients’ perception of treatment efficacy is direct and personal. That is, patients evaluate treatment efficacy based on how well they responded to the treatment. Clinicians’ perception, on the other hand, is an aggregate judgment, but presumably is based on how well the treatment worked for their patient population.

**Figure 1:**
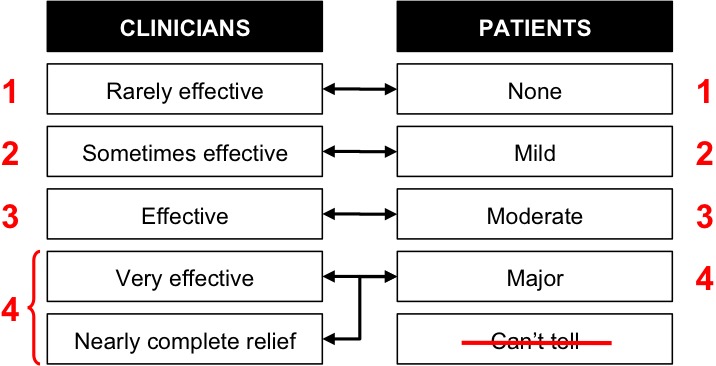
Adjustment of rating scales to enable comparison.

To enable a fair comparison between the two datasets, we made a few adjustments and transformations. PatientsLikeMe provided a dataset for analysis. We started by searching for efficacy data that would match the 56 symptom–treatment pairs from the Forshew and Bromberg study. We then discarded data on any symptom–treatment pair for which patient sample size was smaller than 6 patients. Given that each pair had a different sample size, we converted the frequencies to percentages to facilitate a visual comparison. Finally, we adjusted the rating scales from both sources. The rating scales from the Forshew and Bromberg study and from PatientsLikeMe were composed of five levels. Items rated as “Can’t tell” from the PatientsLikeMe dataset were not included in our comparative analysis because (1) they were not part of an ordinal scale like the 4 other items (ie, none, mild, moderate, or major), and (2) they referred to cases in which patients had not yet formed an opinion. We combined the two top levels in the Forshew and Bromberg study because the last item in the scale, nearly complete relief, was quite small (corresponding to less than 3% of the ratings), and it interrupted the continuity of the scale. The first 4 items in the scale explicitly used the word *effective*. Further, the first two qualifiers (ie, *rarely *and *sometimes*) strongly suggested a rating scale based on frequency. The last item in the scale, however, suggested a rating based on the intensity rather than on the frequency of the effect by using the qualifier *complete*. Figure 1 summarizes the adjustments made to both scales to facilitate a comparative analysis.

## Results

As of March 14, 2010, the ALS community on PatientsLikeMe consisted of 4375 members, of whom 41% were female and 59% were male. Of the 697 members who disclosed their age, 3% were under 30 years old, 10% were between 30 and 39 years old, 24% were between 40 and 49 years old, 29% were between 50 and 59 years old, 26% were between 60 and 69 years old, and 8% were older than 69 years. This gender and age distribution is consistent with that of the general ALS population: ALS occurs more frequently in men than in women with a ratio of approximately 1.5:1 to 2.0:1. It is also a disease of upper middle age with a mean age of onset between 55 and 65 years [12].

### Prevalence of Treatment Usage

After filtering out the symptoms with fewer then 10 respondents from the list of 13 overlapping symptoms, 10 symptoms and 15 symptom–treatment pairs remained. Table 1 shows the number of patients who responded to the question on the treatments prescribed or received for the 10 specific symptoms. Table 2 shows the 15 symptom–treatment pairs. None of the 15 pairs was used by either group more than 50% of the time. In 7 of the 15 pairs, the discrepancy in usage between the two groups was above 10%. In 3 pairs the differences were over 20%: amitriptyline for labile emotional effect; oxybutynin for urinary urgency; and amitriptyline for disturbed sleep.

**Table 1:** Number of clinicians and patients who responded to the question regarding treatments prescribed or received for specific symptoms.

Symptom	Clinicians’ report	Patients’ report
Muscle cramps	31 (51%)	95 (35%)
Sialorrhea	30 (49%)	258 (17%)
Depression	31 (51%)	300 (19%)
Anxiety	29 (48%)	240 (15%)
Spasticity and stiffness	31 (51%)	848 (39%)
Labile emotional effect	28 (46%)	147 (9%)
Urinary urgency	23 (38%)	31 (25%)
Disturbed sleep	31 (51%)	216 (15%)
Fasciculations	20 (33%)	112 (5%)
Constipation	28 (46%)	200 (15%)

**Table 2:** Comparison of percentage of clinicians reporting preferences for specific treatment use in response to a symptom, and patient-reported usage of the same treatments.

Symptom	Treatment	Clinicians’ report	Patients’ report
Muscle cramps	Quinine	30 (35%)	30 (32%)
	Baclofen	16 (19%)	26 (27%)
Sialorrhea	Amitriptyline	32 (36%)	75 (29%)
	Glycopyrrolate	20 (23%)	46 (18%)
Depression	Sertraline	21 (24%)	50 (17%)
	Fluoxetine	16 (18%)	28 (9%)
Anxiety	Lorazepam	16 (24%)	65 (27%)
Spasticity and stiffness	Baclofen	37 (40%)	246 (29%)
	Tizanidine	21 (23%)	42 (5%)
Labile emotional effect	Amitriptyline	30 (50%)	31 (21%)
Urinary urgency	Oxybutynin	22 (46%)	6 (19%)
Disturbed sleep	Amitriptyline	29 (34%)	24 (11%)
	Zolpidem	14 (16%)	71 (33%)
Fasciculations	Gabapentin	10 (20%)	9 (8%)
Constipation	Docusate	13 (16%)	38 (19%)

Of the symptom–treatment pairs, 13 corresponded to US Food and Drug Administration-approved (*indicated*) uses of the drugs; 2 pairs corresponded to uses for which an indication had not been granted (*off-label use*): amitriptyline for labile emotional effect and gabapentin for fasciculations.

### Treatment Efficacy

After eliminating symptom–treatment pairs for which the number of efficacy evaluations was smaller than 10, we were left with 5 pairs. In the following paragraphs we present the results for the perceived efficacy of amitriptyline to treat sialorrhea and baclofen to treat spasticity and stiffness. Results for the remaining 3 pairs are provided in Multimedia Appendix 1.

#### Perceived Efficacy of Amitriptyline to Treat Sialorrhea

**Figure 2:**
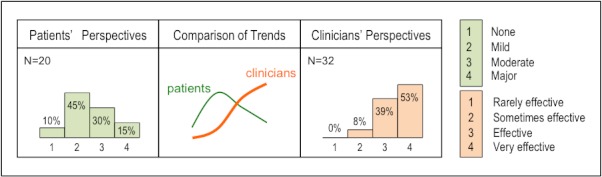
Perceived efficacy of amitriptyline to treat sialorrhea.

Clinicians had a more positive perception of the efficacy of amitriptyline (Figure 2) to treat sialorrhea. Clinician’s ratings for the drug show a clear ascending trend from ratings 2 to 4, with no clinicians’ ratings of 1. Patients’ ratings of amitriptyline show an inverse trend, with a positively skewed distribution.

#### Perceived Efficacy of Baclofen to Treat Spasticity and Stiffness

**Figure 3:**
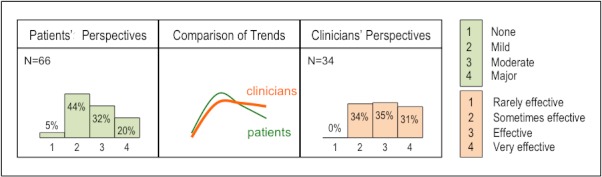
Perceived efficacy of baclofen to treat spasticity and stiffness.

Patients’ ratings for baclofen showed a positively skewed frequency distribution (Figure 3). Clinicians’ ratings were evenly distributed between categories 2 and 4.

## Discussion

We found that clinicians’ and patients’ perceptions of treatment prevalence and efficacy had some differences and could potentially complement each other. Both patients and clinicians nominated a wide range of treatments for ALS symptoms, and there was not always consensus between them regarding the efficacy of each individual treatment. This indicates that more robust empirical data to guide clinical practices in the management of ALS symptoms is required, and the data from the social network may be used to inform practice.

The clinicians perceived treatment efficacy as only marginal for some treatments. Symptoms associated with treatments that received an average ranking of 3 or higher were classified as treatable. Symptoms associated with treatments that scored below that threshold were classified as difficult to treat. Patients sometimes give a more positive ranking and sometimes a more negative ranking. Due to the sample size, we did not statistically test the differences.

Limited studies have been done to assess the value of using social network data for research purposes. We are not aware of prior work that compared data gathered from a social network with data from clinicians. Observing consistencies and inconsistencies between the two data sources in this study, we believe that data gathered from an online social network, especially as it grows in popularity, can be a potential data source of patient perspectives for scientific studies.

### Limitations and Future Studies

While providing valuable data, Forshew and Bromberg also duly acknowledge one limitation in the design of their survey: respondents ranked treatments based on an average ALS patient, which oversimplifies the real issue, given that response to treatment may vary according to the disease stage.

Both clinician and patient cohorts are expected to be heavily skewed by self-selection bias. That is, the sample is less likely to be a good representation of a larger population when it is not completely random. In fact, complete sample randomization is a constant issue in any study that deals with human participants. We do not know the demographics or clinical characteristics of patients treated by the physician participants in the Forshew and Bromberg study. We can assume that the patient population was roughly representative of the general patient population, since Forshew and Bromberg conducted a national survey. Similarly, we have little knowledge of the PatientsLikeMe population’s clinical characteristics. The size of the ALS community on PatientsLikeMe, however, is fairly large, representing roughly 5% of the US ALS population. The age and gender of the PatientsLikeMe ALS population do resemble those of the general ALS population.

Given the very small sample sizes for both datasets, the outcomes regarding treatment efficacy are not as robust as one would wish. However, given that the patient pool of PatientsLikeMe grows daily, we expect that such inaccuracies due to sample size will become increasingly smaller in the case of patient-reported data.

In terms of relative sample size, cohorts of clinicians and patients do not lend themselves to a direct comparison. Patients’ observations are based on their personal experiences and thus are direct and singular. Clinicians’ observations, on the other hand, are indirect and aggregate—that is, they are formed based on data coming from a collection of patients. Further, clinicians obviously possess more extensive clinical knowledge than patients do, which makes clinicians’ observations more systematic. Consequently, studies based on patients’ perspectives are likely to require larger sample sizes than studies based on clinicians’ perspectives.

In this study we compared data from only one survey and one online community of patients. Future studies should consider including more instances of each type of data source, which would compensate for the specific limitations and weaknesses of each data source.

Several authors have called attention to the fact that self-selection bias is a common threat to the internal and external validity in studies involving human participants [12,13]. This threat is further magnified in studies that use measurements based on participants’ perception.

### Conclusions

Our comparative analysis detected significant discrepancies between patients’ and clinicians’ perceptions of treatment prevalence and efficacy. Both data sets have their own limitations, which were partially described in the previous sections. However, if they are properly conjugated, their potential to offer a more complete and accurate picture of ALS symptomatic treatment is likely to increase. As previously mentioned, data validity issues related to sample size in PatientsLikeMe are likely to gradually self-correct as the online community grows.

Assessing and comparing the efficacy of the symptomatic treatment of a complex and rare disease such as ALS is not easy. It is also important that patients’ as well as clinicians’ views be taken into consideration. Drawing a reliable profile of treatment efficacy requires taking into consideration many interacting aspects (eg, disease stage and severity of symptoms) that were not covered in the present study. Nevertheless, pilot studies such as the one described here can pave the way for more robust studies by helping researchers anticipate and compensate for limitations in their data sources and study design.

## Multimedia Appendix 1

Perceived efficacy of other treatments.

## Abbreviations

ALS: amyotrophic lateral sclerosis
