# Supplementary material for: Mining Online Social Network Data for Biomedical Research: A Comparison of Clinicians’ and Patients’ Perceptions About Amyotrophic Lateral Sclerosis Treatments
Source: J Med Internet Res. 2012 Jun 21;14(3):e90. doi: 10.2196/jmir.2127 (PMC3414854; doi:10.2196/jmir.2127)

APPENDIX

Perceived Efficacy of Tizanidine to Treat Spasticity and Stiffness

Figure A1. Perceived efficacy of tizanidine to treat spasticity and stiffness

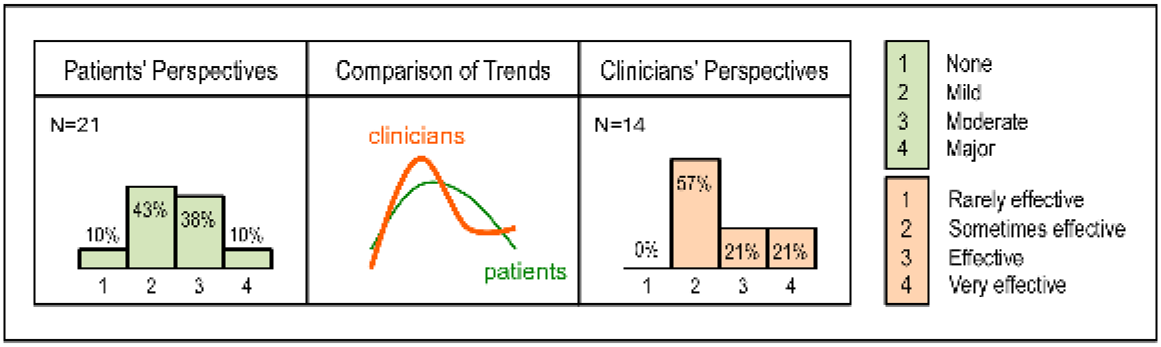

Supplement: Supplementary file 1 [file jmir_v14i3e90_app1.pdf]
